# Rheumatoid arthritis-like features in Hansen disease: A case report

**DOI:** 10.1097/MD.0000000000011590

**Published:** 2018-07-20

**Authors:** Le-Nyu Gao, Bing Zhong, Yong Wang

**Affiliations:** Department of Rheumatology, Southwest Hospital, the Third Military Medical University (Army Military Medical University), Chongqing, China.

**Keywords:** arthritis, Hansen Disease, rheumatoid arthritis

## Abstract

**Rationale:**

Hansen disease is an infectious chronic disease with various clinical manifestations. Its joint performance may easily mimic rheumatoid arthritis.

**Patient concerns:**

We report a case of a 57-year-old woman diagnosed with Hansen disease 10 years ago, who suffered from joints swelling, pain and joints deformities of both hands for 19 years. The skin on the hands showed rashes, thickening, desquamation and chapping, with both thenar muscles atrophy. She also had severe hypoalgesia of the whole body, and morning stiffness for one hour.

**Diagnoses:**

The final diagnosis was joint damage and peripheral neuropathy due to Hansen disease.

**Interventions:**

The patient received neurotrophic treatment instead of anti-rheumatic treatment.

**Outcomes:**

At 1-year follow up, no further aggravation of joint swelling and pain was detected.

**Lessons:**

The correct diagnosis of Hansen disease involving joints depends on the combination of medical history, careful physical examination, and laboratory examination.

## 1 Introduction

Hansen disease is a chronic infectious disease caused by leprosy bacillus, which mainly damages the skin and peripheral nerves, and can also invade visceral and bone joints. Its clinical manifestations are complex, variable, and easily misdiagnosed. Rheumatological manifestations are frequent, although often under-recognized. Occasionally, such patients may present to a rheumatology clinic prior to the diagnosis of Hansen disease.^[1]^ We reported a case of joint damage caused by Hansen disease similar to rheumatoid arthritis (RA).

## 2 Case report

A 57-year-old female, with a history of tuberculosis and diabetes, visited the department of rheumatology in March 2016. She reported 19 years of swelling, pain, and numbness of multiple joints. The pain started on her ankles and feet, with both hands and feet numbness 19 years ago. Joints swelling and pain gradually developed in the metacarpophalangeal joints, proximal interphalangeal joints, wrists, shoulders, and knees, with morning stiffness for 1 hour. She was diagnosed with RA, and the drug treatment was unknown. Ten years after the diagnosis, she developed low back pain, which was more obvious at night than in daytime, and both the low back pain and joint pain were relieved after movement. Three years ago, a diagnosis of “binocular uveitis” was made due to the binocular vision blurring.

**Figure 1:**
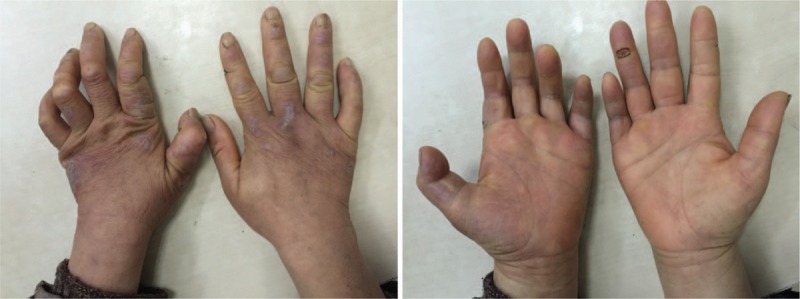
The right 2–5 proximal interphalangeal joints were swollen, left 2–5 proximal interphalangeal joints presented flexion and deformities. The skin on the hands showed rashes, thickening, desquamation and chapping, with both thenar muscles atrophy.

On physical examination, the patient presented heel tenderness, right 2 to 5 proximal interphalangeal joints swelling, left 2 to 5 proximal interphalangeal joints flexion and deformities. The skin on the hands showed rashes, thickening, desquamation and chapping, with both thenar muscles atrophy (Fig. 1). She also had severe hypoalgesia of the whole body, and loss of the external third of the eyebrow. Lumbar spinous process and sacroiliac joints were slightly tender. Bilateral Patrick sign was negative. Bilateral Lasegue test was positive. Modified Schober test was 3 cm. Thoracic expansion was 3 cm. Floor-finger tip distance was 5 cm. Wall-tragus distance was 11 cm.

**Figure 2:**
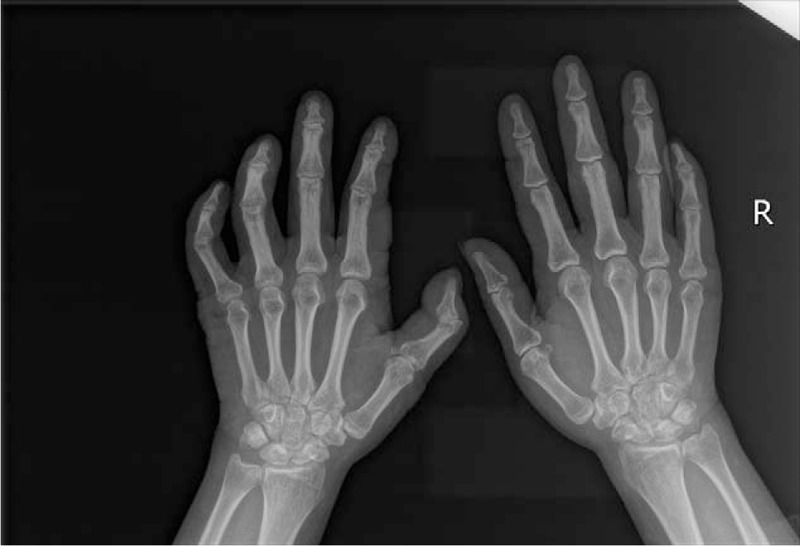
X-ray of the hands: no bone destruction, the bone density of the metacarpophalangeal joints was reduced, the joint space was normal.

**Figure 3:**
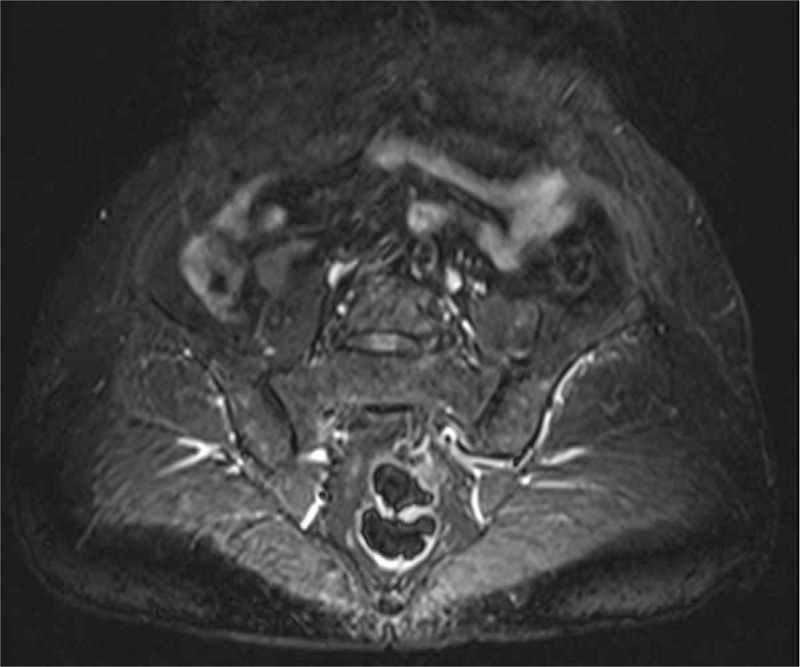
No abnormality was found in the MRI of the sacroiliac joint or the hips.

Initial laboratory test results were normal, including complete blood count, ESR (3 mm/h), CRP (1.7 mg/L), creatinine (44 μmol/L), transaminase (AST 18.9 IU/L, ALT 7.5 IU/L), anti-CCP (<25 RU/mL), anti-MCV (<40 IU/mL), and HLA-B27. RF was 33.90 IU/mL, but RF IgG was<25 IU/mL, RF IgA<25 IU/mL, RF IgM<15 IU/mL. ANA was positive, with speckled pattern, and weakly positive tuberculosis antibody. Electromyogram indicated extensive peripheral neuropathy. Chest computed tomography (CT): small nodules were seen in the lateral segment of the middle lobe of the right lung. X-ray of the hands (Fig. 2): no bone destruction, the bone density of the metacarpophalangeal joints was reduced, the joint space was normal. X-ray of the spine: the curvature of the cervical spine was slightly straight, with lumbar degenerative changes. No abnormality was found in the MRI of the sacroiliac joint or the hips (Fig. 3). Joint ultrasound: synovitis was found in all the metacarpophalangeal joints, proximal interphalangeal joints and bilateral wrists; the right finger flexor tendon and right ulnar tendon showed tenosynovitis.

**Figure 4:**
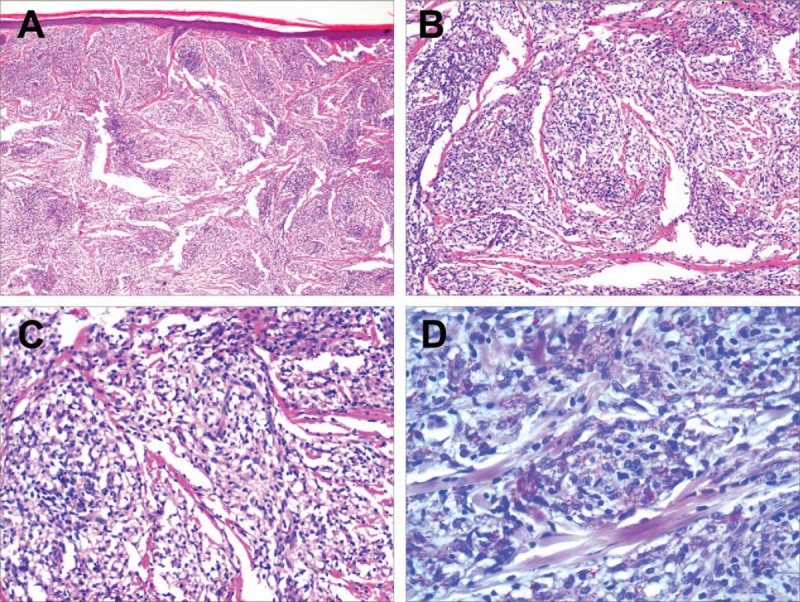
Photomicrograph of a skin biopsy. A, B, There was infiltration zone under the epidermis with mild atrophy and thinning (HE, × 40, 100). **C,** There were a few lymphocytes and many different sizes of histiocyte masses in the dermis. The cytoplasm of histiocytes was lightly stained (HE, × 200). D, Numerous bacilli were found (acid-fast stain, × 400).

The initial diagnosis was RA according to the 2010 ACR/EULAR RA classification criteria ^[2]^ with 4 small joints involvement, low-positive RF, duration of symptoms >6 weeks, a total score of 6. However, this could not explain the systemic extensive peripheral neuropathy and thenar atrophy, and the joint deformities showed no destruction of bone or joint space narrowing in the radiological examination after a course of 19 years, which were inconsistent with RA. A detailed enquiry of the patient's medical history revealed that she had developed diffuse skin nodules on the whole body ten years ago, along with a loss of the external third of the eyebrow. She was diagnosed as lepromatous leprosy after skin biopsy. The biopsy was characterized by infiltration zone under the epidermis, which had mild atrophy and thinning. There were a few lymphocytes in the dermis and many different sizes of histiocyte masses with lightly stained cytoplasm. The appendages were reduced. Numerous bacilli were found by acid-fast stain (Fig. 4). After three years of standardized treatment at the local epidemic prevention station, the skin masses disappeared. One year after drug withdrawal, the symptoms of joint swelling, pain, and numbness reappeared. Given the extensive peripheral neuropathy, chronic specific skin lesions, and previous skin biopsy diagnosis of lepromatous leprosy, the patient was diagnosed as joint damage and peripheral neuropathy due to Hansen disease.

After the diagnosis, the patient underwent two months neurotrophic treatment consisting of mecobalamin 500 μg orally tid and vitamin B1 5 mg orally tid, instead of anti-rheumatic treatment. At the 1-year follow-up, the arthritic symptoms did not disappear, but no further aggravation of joint swelling and pain was detected.

## 3 Discussion

Hansen disease is an infectious chronic disease with a wide range of clinical and serological manifestations. The clinical and serological similarities between patients with Hansen disease and connective tissue disorders may lead to erroneous diagnosis. Hansen disease can resemble autoimmune disorders, including systemic lupus erythematosus or RA.^[3]^ Recognition of rheumatic manifestations in Hansen disease is important.^[4]^ During the Lepra reaction, the patient may show Charcot's arthritis, acute symmetric multi-arthritis, or hand and foot swelling. The typical joints affected are the wrists, small joints of the hands and feet, as well as the knees.^[5]^ The insidious onset of chronic symmetrical poly-arthritis may be easily misdiagnosed as RA, independent or arthritis-related neuropathy or tenosynovitis. The prevalence of Hansen disease-related arthritis is described in up to 75% of these patients, which can be accompanied by or precede skin lesions and may be the only manifestation,^[5–7]^ and it is the third commonest after dermatological and neurological manifestations.^[8]^ The bone joint damage caused by Hansen disease is divided into three categories: direct bone damage, mainly in leprosy osteitis and expansive bone destruction; secondary bone joint damage due to neurovascular disease, mainly in bone resorption and Charcot's joint; osteoporosis.^[9]^ The cause of joint damage is unknown, and may be associated with Lepra reaction (type I or type II), which is related to the direct infiltration of the synovium and peripheral neuropathy.^[6]^

Hansen disease may show various auto-antibodies such as ANA, anti-dsDNA, anti-cardiolipin antibodies, anti-CCP, and RF.^[4,10]^ The presence of ANA has been reported in 0% to 37.5% of the patients, usually at a low titer, and with speckled and homogenous patterns.^[11]^ Anti-CCP and RF have significant diagnostic value for RA and have high positive rate, which can be used as the clinical diagnostic evidence of RA. However, they can also be positive in some Hansen disease patients. Dionello et al^[10]^ reported 97 patients diagnosed as Hansen disease (71.1% patients with neuropathy, 35.1% with arthritis) who underwent anti-CCP and RF detection, the detection rates were 9.3% and 41.2%, respectively. Another Mexican adult leprosy study^[12]^ reported significant anti-CCP in 5.9% patients and RF in 16.8% patients. Moreover, RF positivity is not the only standard for the diagnosis of RA. In addition to RA, RF positivity can also be found in Sjogren syndrome, systemic lupus erythematosus, infection (such as hepatitis B, subacute bacterial endocarditis), and even in some normal cases. Hence, anti-CCP and RF are not reliable in the differential diagnosis of joint damage in Hansen disease and RA, but the clinical manifestations along with x-ray are the key to differential diagnosis. Therefore, a correct diagnosis should be made after a comprehensive assessment of the patient's clinical, radiological and laboratory tests.

However, RA could co-exist with Hansen disease. Chronic arthritis was seen in an US-based study of Hansen disease,^[13]^ in which 18 of 261 subjects with both Hansen disease on skin biopsy and chronic arthritis were identified by the National Hansen Disease Program between 2001 and 2015. Patients were symptomatic with arthritis for a median of 5.3 years before Hansen disease diagnosis and 11 of them were diagnosed with RA before Hansen disease diagnosis, of which 10 were seronegative RA. A 77-year-old Japanese man with Hansen disease and an 11-year history of shoulder and back pain had lost all ten fingers; a small nodular lesion was detected adjacent to the pleura in the right lower lobe (S10) on chest CT. A lung biopsy was performed under thoracoscopic guidance. Histological examination of the resected nodule with colliquative necrosis revealed palisading granulomas with multinucleated giant cells and plasma cell infiltration, with formation of lymphoid follicles. Since serum levels of both anti-MMP3 and anti-CCP antibodies were elevated, RA with rheumatoid lung was diagnosed.^[14]^

Although the patient in our case had joints swelling, pain and joints deformities of both hands with RF 33.90 IU/mL (positive), but RF IgG<25 IU/mL, RF IgA<25 IU/mL, RF IgM<15 IU/mL, anti-CCP<25 RU/mL, anti-MCV<40 IU/mL, CRP 1.7 mg/L, and ESR 3 mm/h (all negative). Articular surface cystic changes, invasive bone destruction, joint space narrowing, joint fusion, and dislocation are the typical manifestations of RA in the x-ray. However, no narrowing of joint space or bone destruction was found in this patient's x-ray of the hands after 19 years of joint involvement. The joint damage was thought to be caused by leprosy neuropathic arthritis. The final diagnosis was Hansen disease due to the extensive peripheral neuropathy, chronic specific skin lesions, and previous skin biopsy, instead of RA or RA co-existing with Hansen disease. After the diagnosis, the patient underwent 2 months of neurotrophic treatment consisting of mecobalamin 500 mg orally tid and vitamin B1 5 mg orally tid. Although the treatment did not improve the joint symptoms or significantly reduce the numbness, the joint symptoms did not progress at the later stage.

## 4 Conclusions

Hansen disease was a common infectious disease before liberation and in the early days of new China. It was once eliminated by the efforts of the clinicians. In recent years, there are sporadic cases and related reports.^[15]^ The clinical manifestations of leprosy are diverse, so the diagnosis may be a greater challenge in non-endemic countries because of low awareness.^[16]^ Rheumatological presentations of leprosy may mimic RA, spondyloarthropathy or vasculitis.^[1]^ Clinicians need to enquire about the patient's history, conduct detailed physical examination of the whole body, and combine the laboratory examination with a comprehensive assessment to make the correct diagnosis. The possibility of Hansen disease should be considered when patients have joint symptoms or positive RF complicated with skin lesions and peripheral neuropathy. Increased awareness may avoid delay in diagnosis or misdiagnosis.

## Author contributions

**Writing – original draft:** Le-Nyu Gao.

**Writing – review & editing:** Bing Zhong, Yong Wang.
